# Health-related quality of life, depression, and self-esteem in adolescents with leprosy-affected parents: results of a cross-sectional study in Nepal

**DOI:** 10.1186/1471-2458-13-22

**Published:** 2013-01-10

**Authors:** Nobuko Yamaguchi, Krishna C Poudel, Masamine Jimba

**Affiliations:** 1 Department of Nursing, School of Health and Social Services, Saitama Prefectural University, 820 Sannomiya, Koshigaya, Saitama, 343-8540, Japan; 2 Department of Public Health, School of Public Health and Health Sciences, University of Massachusetts Amherst, 316 Arnold House, 715 North Pleasant St, Amherst, MA, 01003-9304, USA; 3 Department of Community and Global Health, Graduate School of Medicine, The University of Tokyo, 7-3-1 Hongo, Bunkyo-ku, Tokyo, 113-0033, Japan

**Keywords:** Adolescents, Leprosy-affected parents, Quality of life, Mental health, Nepal

## Abstract

**Background:**

Leprosy is a chronic infectious disease that has an impact on the Health-Related Quality of Life (HRQOL) of sufferers as well as their children. To date, no study has investigated the effects of parental leprosy on the well-being of adolescent children.

**Methods:**

A cross-sectional study was conducted in the Lalitpur and Kathmandu districts of Nepal. Adolescents with leprosy-affected parents (n = 102; aged 11–17 years) and those with parents unaffected by leprosy (n = 115; 11–17 years) were investigated. Self-reported data from adolescents were collected using the Kinder Lebensqualität Fragebogen (KINDL^R^) questionnaire to assess HRQOL, the Center for Epidemiological Studies-Depression Scale (CES-D), and the Rosenberg Self-esteem Scale (RSES). Analysis of covariance (ANCOVA) was used to compare scores between the two groups. Multiple regression analysis was conducted to explore the determinants of HRQOL for adolescents with leprosy-affected parents.

**Results:**

ANCOVA revealed that the KINDL^R^ and RSES scores were significantly lower among adolescents with leprosy-affected parents compared with unaffected parents. However, the scores of “Friends” and “School” subscales of KINDL^R^ were similar between the two groups. The CES-D score was significantly higher among adolescents with leprosy-affected parents than for adolescents with unaffected parents. The KINDL^R^ scores for adolescents with both parents affected (n = 41) were significantly lower than the scores for those with one parent affected (n = 61). Multiple regression analysis revealed that adolescents with leprosy-affected parents who had higher levels of depressive symptoms were more likely to have lower KINDL^R^ scores. A similar result was seen for adolescents where both parents had leprosy.

**Conclusions:**

Adolescents with leprosy-affected parents had higher levels of depressive symptoms, lower levels of self-esteem, and lower HRQOL compared with adolescents whose parents were unaffected by leprosy. Thus, mental health support programs might be necessary for adolescents with leprosy-affected parents, particularly for adolescents where both parents are leprosy-affected. Further studies with larger sample sizes are necessary to draw decisive conclusions.

## Background

Leprosy is a chronic infectious disease that typically affects the skin and peripheral nerves. According to the World Health Organization (WHO), the South East Asia region accounts for 59.2% of leprosy cases worldwide [1]. The WHO has developed a global strategy for 2011–2015 that aims to reduce the burden of leprosy, and improve treatment and early detection of new cases. For rehabilitation, patients need to be provided with high-quality care to reduce the number of disabilities, and to deal with the socioeconomic stigma associated with leprosy [2].

Nepal is one of many South Asian countries where leprosy is endemic. Leprosy patients are stigmatized in Nepalese society, particularly those with visual deformities [3]. The family members of patients also experience limitations and restrictions in their social life [4,5]. Although intensive rehabilitation of psychological and socioeconomic support networks of leprosy-affected people and their families is increasing, these individuals have to deal with a poor quality of life and an ongoing struggle against stigma [6-8]. Nepalese people with a Hindu cultural background generally believe that leprosy is a result of ritually unclean behavior or a form of punishment for a misdeed (or misdeeds) in a former life [4]. However, public awareness of leprosy is increasing because of the spread of health services treating this affliction, improving literacy rates, and because of a change in attitude regarding traditional beliefs in some regions of Nepal [9].

Leprosy has been demonstrated to have an impact on Health-related Quality of Life (HRQOL) and the mental health status of affected individuals [10-12]. The self-esteem as well as physical and emotional well-being of leprosy patients and their families are likely to be affected. It has also been postulated that the school lives of adolescent children whose parents suffer from leprosy are also affected [13-16].

Parental diseases are known to influence the HRQOL or well-being of adolescents and children. Common diseases where this has been investigated include Parkinson’s disease [17], multiple sclerosis [18], mental illness [19], and HIV/AIDS [20]. Similar to observations seen for other chronic diseases, the mental health of adolescent children, whose parents suffer from leprosy, can be adversely affected. However, very little research regarding this topic has been published. Previously, the importance of epidemiological studies has been emphasized for leprosy-affected children in endemic areas [21,22], but not for adolescents with leprosy-affected parents.

The objective of this study was to investigate the impact of parental leprosy on the well-being of adolescents. We hypothesized that HRQOL, depression, and self-esteem scores between adolescents with and without leprosy-affected parents would be different. Additionally, there might be an apparent difference in HRQOL, depression, and self-esteem scores between adolescents with one leprosy-affected parent and those with both parents affected. We also hypothesized that demographic characteristics, depression, and self-esteem would correlate with HRQOL scores for adolescents with leprosy-affected parents.

## Methods

This was a cross-sectional study among adolescents with and without leprosy-affected parents. In this study, a leprosy-affected parent was defined as the parent of an adolescent that had previously undergone, or was currently undergoing, treatment for leprosy.

### Participants

This study was conducted in the Lalitpur and Kathmandu districts of Nepal. Adolescents of leprosy-affected parents were recruited from the National Leprosarium of Lalitpur and local hostels for school-aged children. Only school-age children with leprosy or leprosy-affected parents reside in these hostels. Most children in these hostels were from the National Leprosarium while others were from the Central Region of Nepal. Adolescents who live in hostels return to their homes on holidays. The leprosarium accommodates approximately 250 leprosy-affected people and their families. Institutional care in the leprosarium is confined to a basic supply of food, clothing, benefit money, and medical care [23].

The inclusion criterion in the present study was age between 11 and 17 years. The exclusion criteria for adolescents were: (i) having received a diagnosis of leprosy or any other chronic disease within 2 months prior to the fieldwork; and (ii) not willing to participate in the study.

In total, 135 participants with leprosy-affected parents and an equal number of adolescents with parents unaffected by leprosy as controls were selected. Informed consent sheets were distributed to all participants and parents’ signatures were collected to verify parental consent. An adolescent was only included in the study if a parent of the adolescent provided written informed consent. Thirty-three adolescents with leprosy-affected parents and 20 adolescents without leprosy-affected parents were excluded, as they were not willing to participate in this study. Eventually, 102 adolescents of leprosy-affected parents and 115 adolescents with parents unaffected by leprosy were enrolled in this study. Of the 102 adolescents of leprosy-affected parents, 78 were recruited from hostels and 24 from the leprosarium. Adolescents of leprosy-unaffected parents were recruited from two schools in the study area. The education system of Nepal consists of 5 years of primary education, 3 years of lower secondary education, 2 years of secondary education, and 2 years of higher secondary education.

The study protocol was approved by the Ethical Committees of The University of Tokyo, Japan, and the Nepal Health Research Council, Nepal.

### Measures

Measures included demographic characteristics and assessments of HRQOL, depression, and self-esteem. HRQOL was assessed using the Kinder Lebensqualität Fragebogen (KINDL^R^), which covers “Physical Well-Being”, “Emotional Well-Being”, “Self-Esteem”, “Family”, “Friends”, and “School”. The psychometric results of this scale have a high reliability and validity among children and adolescents using various languages [24,25]. KINDL^R^ offers 24 items referring to the past week with 5-point Likert-scales (“Never” to “All the time”), with 11 items being reverse-coded. The scores range from 0 to 100, where higher scores indicate higher levels of HRQOL among teenage adolescents. A Nepalese version of KINDL^R^ has internal consistency, reproducibility, responsiveness, interpretability, and discriminant validity [26].

The depressive status of participants was measured using the Center for Epidemiological Studies-Depression Scale (CES-D). This scale is one of the most frequently used standardized measurements in primary depression screening, and is widely used for clinical and research purposes among general populations [27]. CES-D offers 20 items with 4-point Likert-scales (“Rarely or none of the time” to “Most or all of the time”), with 4 items being reverse-coded. For each statement, the subject chooses the response that best describes how often they felt or behaved this way during the past week. The scores range from 0 to 60, with higher scores indicating higher levels of depressive mood.

Self-esteem was examined using the Rosenberg Self-Esteem Scale (RSES). Globally, RSES is the most widely used scale to measure self-esteem among adolescents [28]. RSES offers 10 items with 4-point Likert-scales (“Strongly agree” to “Strongly disagree”), with 5 items being reverse-coded. Participants rated the extent to which they have experienced each symptom over the past week. The scores range from 0 to 30, with higher scores indicating higher levels of self-esteem.

### Data collection

Data were collected from March to May 2008 using the self-administered questionnaire survey. All participants completed the questionnaires by themselves. Data were collected from the Leprosarium by conducting a door-to-door survey based on a resident’s list. The questionnaire survey was carried out at the hostels and data collected during the school breaks. In schools, the questionnaires were administered after school. All participants answered the questionnaire without the intervention of their guardian or teachers.

### Data analysis

The demographics between adolescents with and without leprosy-affected parents were compared. Significant differences of categorical variables [gender, category (years), and school grade] and of continuous variables of age were tested by Chi-square tests and *t*-tests, respectively. The score distributions of KINDL^R^, CES-D, and RSES between adolescents with and without leprosy-affected parents were measured and compared between the two groups. The analysis of covariance (ANCOVA) was used with grade as a factor, since a significant difference in this variable was detected. Grade was a dichotomous variable with two values (1–5, 6–12). For adolescents with leprosy-affected parents, the total and the subscale scores of KINDL^R^, CES-D, and RSES were compared between adolescents having one parent or both parents affected with leprosy. The *t*-test was performed for the differences between the mean values of these scores.

Cohen’s effect size was used, taking the differences between the two means divided by the standard deviation of the scores. Effect sizes were interpreted as small (0.2), medium (0.5), and large (above 0.8) [29].

To explore the determinants of HRQOL for adolescents with leprosy-affected parents, multiple regression analysis with the total score of KINDL^R^ as a dependent variable were conducted. Age, gender, parental status (one or both parents affected with leprosy), residence of participants (leprosarium or hostels), and CES-D total scores were selected as independent variables. RSES scores and grade were excluded from the model because of the multicollinearity with CES-D and age (Variance Inflation Factor [VIF]: > 2.0), respectively. All statistical analyses were performed using SPSS ver.16.0 (SPSS Inc., Chicago, IL).

## Results

Table 1 shows the demographic characteristics of the study participants. The mean ages of 102 adolescents with leprosy-affected parents and 115 adolescents without leprosy-affected parents were 13.8 years and 14.5 years, respectively. Of the total, 36.3% of adolescents of parents affected with leprosy were in primary school while 63.7% were in secondary school. Significant differences in mean age, age category (years old), and grade were identified between these two groups.

**Table 1:** Demographic characteristics of the participants

		**Adolescents with parents affected by leprosy**^**1)**^**(n = 102)**		**Adolescents with parents unaffected by leprosy**^**2)**^**(n = 115)**		**p value**^**3)**^
		**n**	**(%)**	**n**	**(%)**	
Gender						
	Boys	50	(49.0)	56	(48.7)	.962
	Girls	52	(51.0)	59	(51.3)	
Age						
	Mean (SD)	13.8	(1.6)	14.5	(1.3)	.002
Category (years)						
	11	6	(5.9)	3	(2.6)	.003
	12	23	(22.5)	6	(5.2)	
	13	15	(14.7)	15	(13.0)	
	14	21	(20.6)	30	(26.1)	
	15	17	(16.7)	37	(32.2)	
	16	15	(14.7)	19	(16.5)	
	17	5	(4.9)	5	(4.3)	
School grade ^4)^						
	1-5	37	(36.3)	0	(0.0)	<.001
	6-12	65	(63.7)	115	(100.0)	
Residence of participants						
	Hostel	78	(76.5)			
	Leprosarium	24	(23.5)			
Participants with one or both parents affected by leprosy						
	One parent	61	(59.8)			
	Both parents	41	(40.2)			

^1)^ The study was carried out in the hostels and leprosarium where they live.

^2)^ A school-based study was carried out in two schools.

^3)^ Gender, category (years), and school grade were analyzed by Chi- square test. Age was analyzed by *t-*test.

^4)^ The education system of Nepal consists of 5 years of primary education , 3 years of lower secondary education, 2 years of secondary education , and 2 years of higher secondary education.

Among adolescents with leprosy-affected parents, 78 were living in hostels and 24 were cohabiting with parents in the leprosarium. Among these participants, 61 adolescents had one parent with a history of leprosy (father; 53, mother; 8) and 41 had two parents with a history of leprosy.

Table 2 shows the total and subscale scores and comparative test results for KINDL^R^, CES-D, and RSES scores of adolescents with and without leprosy-affected parents. The means of total KINDL^R^ scores among adolescents with leprosy-affected parents and those without were 55.9 (SD 13.1) and 63.0 (SD 9.9), respectively. The effect size was medium (0.63) in magnitude between the two groups. Subscale scores were significantly lower among the participants with leprosy-affected parents compared with those without except for the subscales of “Friends” and “School”. The “Self-Esteem” subscale had the lowest score and “Friends” subscale had the highest score among adolescents with leprosy-affected parents. The lowest and highest subscales in KINDL^R^ among adolescents with parents unaffected by leprosy were “School” and “Family”, respectively. The effect sizes of subscales between the two groups were large to small (1.04 to 0.06).

**Table 2:** Comparison of the KINDL^R,^ CES-D, and RSES scores for adolescents with leprosy-affected parents and adolescents with parents unaffected by leprosy

	**Scales**	**Adolescents with parents affected by leprosy (n = 102)**		**Adolescents with parents unaffected by leprosy (n = 115)**		***F***^**1)**^	***p *value**	**Effect size**^**2)**^
		**Mean**	**SD**	**Mean**	**SD**			
KINDL^R 3)^								
	Total Score	55.9	13.1	63.0	9.9	29.25	<.001	0.63
	Physical Well-Being	51.2	14.5	59.2	15.4	10.38	.001	0.53
	Emotional Well-Being	50.5	14.2	63.9	17.1	43.56	<.001	0.86
	Self-Esteem	47.5	19.2	57.3	20.1	11.98	.001	0.49
	Family	58.6	18.5	76.1	14.9	67.27	<.001	1.04
	Friends	72.0	19.6	67.5	16.8	0.07	.794	0.24
	School	55.5	23.5	54.4	15.8	0.65	.421	0.06
CES-D^4)^		14.3	7.5	11.3	7.8	8.89	.003	0.40
RSES^5)^		19.2	6.0	22.8	4.7	22.83	<.001	0.66

^1)^ Analysis of covariance (ANCOVA) was used with grade as a factor.

^2)^ Effect sizes as used in analyses were calculated by taking the differences between the two means of the adolescents with and without leprosy-affected parents, divided by the standard deviation.

^3)^ The scores range from 0 to 100, where higher scores indicate higher levels of HRQOL.

^4)^ The scores range from 0 to 60, where higher scores indicate higher levels of depressive mood.

^5)^ The scores range from 0 to 30, where higher scores indicate higher levels of self-esteem.

The means of the total scores of CES-D and RSES were compared between both groups. The CES-D score among adolescents with leprosy-affected parents was significantly higher than for adolescents without leprosy-affected parents. The mean of the total RSES score was significantly lower among adolescents with leprosy-affected parents than among those without leprosy-affected parents. Cronbach’s alpha values of KINDL^R^, CES-D, and RSES were 0.81, 0.80, and 0.70, respectively.

Table 3 shows comparisons of KINDL^R^, CES-D, and RSES scores in adolescents with one parent and both parents affected with leprosy. A *t*-test was performed to determine the mean difference between the two groups owing to smaller sample sizes. Means of total scores of KINDL^R^ for adolescents with one parent or both parents affected with leprosy were 59.6 (SD 11.0) and 50.4 (SD 14.0), respectively. Overall, the subscale scores of adolescents with both parents affected were significantly lower than for those with one parent affected, except for the “Physical Well-Being” subscale. The effect sizes between the two groups were medium to small (0.74 to 0.11). CES-D and RSES showed no significant differences between the two subgroups.

**Table 3:** Comparison of the KINDL^R,^ CES-D, and RSES scores for adolescents with one and both parents affected by leprosy

	**Scales**	**Adolescents with one parent affected by leprosy (n = 61)**		**Adolescents with both parents affected by leprosy (n = 41)**		***p *value**^**1)**^	**Effect size**^**2)**^
		**Mean**	**SD**	**Mean**	**SD**		
KINDL^R^							
	Total Score	59.6	11.0	50.4	14.0	<.001	0.74
	Physical Well-Being	51.9	15.0	50.3	13.8	.602	0.11
	Emotional Well-Being	53.1	12.9	46.7	15.4	.024	0.45
	Self-Esteem	51.8	18.1	41.2	19.3	.006	0.57
	Family	62.4	15.6	52.9	21.2	.010	0.52
	Friends	77.3	16.5	64.0	21.2	.001	0.70
	School	61.1	21.2	47.1	24.5	.003	0.61
CES-D		13.6	7.6	15.4	7.2	.229	0.25
RSES		19.7	5.9	18.5	6.3	.345	0.19

^1)^*t*-test was used for significant differences between adolescents with one and both parents affected by leprosy.

^2)^ Effect sizes as used in the analyses were calculated by taking the differences between the two means of the adolescents with one and both parents affected by leprosy , divided by the standard deviation.

Multiple regression analysis was conducted to identify determinants of HRQOL (Table 4). Adolescents with higher levels of depressive symptoms and those having two leprosy-affected parents were more likely to have lower KINDL^R^ scores. In the multivariable regression analysis, the value for adjusted R-Squared was 0.44. The value of R-Squared is a quantitative measure of how well the independent variables account for the outcome. In this study, CES-D and parental leprosy status were more important HRQOL predictors for adolescents with leprosy-affected parents.

**Table 4:** Multiple regression analysis of the KINDL^R^ among adolescents with leprosy-affected parents (n = 102)

**Variables**	**KINDL**^**R**^**total scores**	**95% Confidence Interval**	
	**β**^**1)**^	**Lower**	**Upper**
Age	0.07	−0.91	2.12
Gender ^2)^	−0.06	−5.41	2.18
Participants with one or both parents affected by leprosy^3)^	−0.29^**^	−11.39	−3.75
Residence of participants^4)^	−0.07	−6.65	2.20
CES-D	−0.54^**^	−1.21	−0.69

^1)^ Standard multiple regression analysis was used to explore the determinants of the HRQOL for the adolescents with leprosy-affected parents. R-Squared = 0.46, adjusted R-Squared = 0.44,^**^p < .001.

^2)^ Gender (0 = girls, 1 = boys).

^3)^ Participants with one or both parents affected by leprosy (0 = one parent , 1 = both parents).

^4)^ Residence of participants (0 = leprosarium, 1 = hostel).

## Discussion

The aim of the present study was to investigate the impact of parental leprosy on the well-being of adolescent children. The study found that adolescents with leprosy-affected parents had higher levels of depressive symptoms and lower levels of self-esteem and HRQOL. Our results have important implications for implementing mental health programs for adolescents with leprosy-affected parents who currently have limited access to such programs.

In this study, one of the inclusion criteria of adolescents with leprosy-affected parents was not having chronic diseases. The “Physical Well-Being” score of the KINDL^R^ was lower for adolescents with leprosy-affected parents than for those with unaffected parents. Adolescents with leprosy-affected parents are vulnerable to the onset of leprosy [30] and thus, they might worry about contracting the disease despite being in good health and having regular health check-ups.

Adolescents with leprosy-affected parents had lower “Emotional Well-Being” and “Self-Esteem” scores than those of general adolescents. Self-esteem is strongly related to emotional well-being and is an emotional component of personal qualities and competencies. It is generally related to how well or poorly individuals feel about themselves [28]. Leprosy-affected people and their family members are often excluded from social participation at the community level [4,5,7-9]. Such experiences of adolescents with leprosy-affected parents might contribute to their low self-esteem and poor emotional state. Over 90% of students in public schools in the study area come from the poorest quintiles in Nepal [31]. Thus, low self-esteem of the participants in this study might be due to the poverty of their families. Adolescents are highly susceptible to the impact of family events such as poverty, unemployment, and other adverse social circumstances [32].

The KINDL^R^ scores might vary according to these participants’ background. Many adolescents of leprosy-affected parents cannot go to school because of the poor state of the parents’ economic situation [15]. Thus, there is a need to deal with such issues and further enhance the educational environment for these adolescents.

The “Friends” and “School” subscale scores were not significantly different between adolescents with and without leprosy-affected parents. In the hostels or the leprosarium, adolescents stay with other primary stakeholders of leprosy. It is unlikely that the adolescents are discriminated against by adolescents of a similar status. A previous study [33] suggested that the partnerships formed within minority groups promote the strength of solidarity. In addition, fieldwork for this study was conducted soon after school final examinations. In Nepal, such examinations are generally stressful because students might fail and be required to repeat the same grade [34]. This situation might have contributed to the low levels of school subscale scores among the studied adolescents.

Mental health studies in developed and developing countries show that between 10–25% of children and adolescents suffer from a mental health problem [35]. The high prevalence of depressive symptoms among adolescents with parents unaffected by leprosy in this study might be due to their economic backgrounds. Mental health programs are difficult to access for most of the population in Nepal.

The KINDL^R^ total and subscale scores of adolescents with two parents affected with leprosy were significantly different from those with only one parent affected. In addition, the presence of depression and having two parents affected with leprosy significantly affected adolescents’ HRQOL. A previous study reported a similar result where the risk of mental health problems for adolescents was greater when both parents had mental health problems than when a single parent had a mental health problem [36]. Adolescents with leprosy-affected parents might be involved in daily household activities including caring for family members as they grow up [17], especially when both parents are affected with leprosy-related disabilities. Thus, the burden of leprosy and related social problems might be more severe among adolescents with two parents affected with leprosy compared with those with only one parent affected. The results of the present study may be helpful in evaluating the environment of adolescents with leprosy-affected family members and for assessing the range of interventions that might be appropriate.

Our results suggest the need for implementing mental health programs for adolescents with leprosy-affected parents, in particular, those adolescents with both parents affected with leprosy. Moreover, the programs should be designed to reduce or prevent stigma among adolescents with leprosy-affected parents. Most of the patients and their family members, as primary stakeholders in leprosy, are also vulnerable to public stigmatization and misinformation that causes fear or anxiety. Such programs should aim to reduce depressive symptoms and improve self-esteem and subjective well-being among adolescents with leprosy-affected parents, and help them to cope with their parents’ disease [37].

One limitation of our study is the small sample size, and it may not be possible to generalize our results to all adolescents with leprosy-affected parents. Thus, the suggested interventions may not be definitive in Nepal or other countries. However, it is difficult to recruit sufficient participants to adequately represent the target population, because home-based treatment of leprosy patients is common in Nepal as patients want to conceal their disease from the local community. Nevertheless, we recruited participants residing either in the leprosarium or in hostels. The response rate of participants in the study was 75% (102/135). Therefore, our results are applicable to adolescents residing in similar institutions.

Our study has another limitation. The CES-D is a self-rating instrument to identify depressive symptoms during the previous week, and is not a diagnostic tool to identify depression administered by a suitably trained professional.

## Conclusions

Adolescents with leprosy-affected parents had higher levels of depressive symptoms and lower levels of self-esteem and HRQOL compared with those without leprosy-affected parents. Further study with a larger sample size is necessary to confirm our conclusions. Our results suggest that mental health support programs are necessary for adolescents with leprosy-affected parents, in particular, for adolescents with two parents affected with leprosy.

## Abbreviations

HRQOL: Health-related Quality of Life; KINDL: Kinder Lebensqualität Fragebogen; CES-D: Center for Epidemiological Studies-Depression Scale; RSES: Rosenberg Self-esteem Scale.

## Competing interests

The study was supported by Saitama Prefectural University, Saitama, Japan. The authors declare that they have no conflict of interest.

## Authors’ contributions

NY conducted the survey and drafted the initial manuscript. KCP and MJ supervised the study. KCP and MJ helped in the statistical analysis and with manuscript revision. All the authors have read and approved this submission.

## Pre-publication history

The pre-publication history for this paper can be accessed here:

http://www.biomedcentral.com/1471-2458/13/22/prepub

## References

[B1] World Health Organization Leprosy update, 2011 Wkly Epidemiol Rec 2011 86 389 40021887885

[B2] World Health Organization Global leprosy situation, 2010 Wkly Epidemiol Rec 2010 85 337 348 20830851

[B3] Van Brakel WH Anderson AM Mutatkar RK Bakirtzief Z Nicholls PG Raju MS Das-Pattanayak RK The Participation Scale: measuring a key concept in public health Disabil Rehabil 2006 28 193 203 10.1080/09638280500192785 16467054

[B4] de Stigter DH de Geus L Heynders ML Leprosy: between acceptance and segregation. Community behavior towards persons affected by in eastern Nepal Lepr Rev 2000 71 492 498 11201904 10.5935/0305-7518.20000051

[B5] Nicholls PG Bakirtzief Z Van Brakel WH Das-Pattanaya RK Raju MS Norman G Mutatkar RK Risk factors for participation restriction in leprosy and development of a screening tool to identify individuals at risk Lepr Rev 2005 76 305 315 16411510

[B6] Brandsma JW Harijan T Wagle P Baxter KA Partnership for rehabilitation: looking beyond impairments Indian J Lepr 2008 80 19 29 19295118

[B7] Cross H Choudhary R STEP: an intervention to address the issue of stigma related to leprosy in Southern Nepal Lepr Rev 2005 76 316 324 16411511

[B8] Heijnders ML The dynamics of stigma in leprosy Int J Lepr Other Mycobact Dis 2004 72 437 447 10.1489/1544-581X(2004)72<437:TDOSIL>2.0.CO;2 15755198

[B9] Varkevisser CM Lever P Alubo O Burathoki K Idawani C Moreira TM Patrobas P Yulizar M Gender and leprosy: case studies in Indonesia, Nigeria, Nepal and Brazil Lepr Rev 2009 80 65 76 19472853

[B10] Mankar MJ Joshi SM Velankar DH Mhatre RK Nalgundwar AN A Comparative study of the quality of life, knowledge, attitude and belief about leprosy disease among leprosy patients and community members in Shantivan Leprosy Rehabilitation Centre, Nere, Maharashtra, India J Glob Infect Dis 2011 3 378 382 10.4103/0974-777X.91063 22224003 PMC3249995

[B11] Lustosa AA Nogueira LT Pedrosa JI Teles JB Campelo V The impact of leprosy on health-related quality of life Rev Soc Bras Med Trop 2011 44 621 626 10.1590/S0037-86822011000500019 22031079

[B12] Tsutsumi A Izutsu T Islam AM Maksuda AN Kato H Wakai S The quality of life, mental health, and perceived stigma of leprosy patients in Bangladesh Soc Sci Med 2007 64 2433 2453 10.1016/j.socscimed.2007.02.014 17382441

[B13] Ebenso B Ayuba M ‘Money is the vehicle of interaction’: Insight into social integration of people affected by leprosy in northern Nigeria Lepr Rev 2010 81 99 110 20825114

[B14] Scott J The psychosocial needs of leprosy patients Lepr Rev 2000 71 486 491 11201903 10.5935/0305-7518.20000050

[B15] Saylan T Karadeniz A Iyier N Soydan M Pamuk D A scholarship project for the children of leprosy patients in Turkey Lepr Rev 2000 71 212 216 10920616 10.5935/0305-7518.20000023

[B16] Kopparty SNM Kurup AM Sivaram M Problems and coping strategies of families having patients with and without deformities Indian J Lepr 1995 67 133 152 8537702

[B17] Schrag A Morley D Quinn N Jahanshahi M Impact of Parkinson’s disease on patients’ adolescent and adult children Parkinsonism Relat Disord 2004 10 391 397 10.1016/j.parkreldis.2004.03.011 15465394

[B18] Morley D Selai C Schrag A Jahanshahi M Thompson A Adolescent and adult children of parents with Parkinson’s disease: incorporating their needs in clinical guidelines Parkinsons Dis 2011 2011 951874 Epub 2011 Jun 12 21766002 10.4061/2011/951874 PMC3135087

[B19] Giannakopoulos G Dimitrakaki C Pedeli X Kolaitis G Rotsika V Ravens-Sieberer U Tountas Y Adolescents’ wellbeing and functioning: relationships with parents’ subjective general physical and mental health Health Qual Life Outcomes 2009 7 100 10.1186/1477-7525-7-100 20003508 PMC2804705

[B20] Xu T Wu Z Rou K Duan S Wang H Quality of life of children living in HIV/AIDS-affected families in rural areas in Yunnan, China AIDS Care 2010 22 390 396 10.1080/09540120903196883 20390520 PMC3097382

[B21] Sachdeva S Amin SS Khan Z Sharma PK Bansal S Childhood leprosy: lest we forget Trop Doct 2011 41 163 165 10.1258/td.2011.100477 21586510

[B22] Madarasingha NP Senaviratne JK A study of household contacts of children with leprosy Ceylon Med J 2011 56 112 114 22164748 10.4038/cmj.v56i3.3602

[B23] Malla OK Brandt F Anten JGF Ocular findings in leprosy patients in an institution in Nepal (Khokana) Br J Ophthalmol 1981 65 226 230 10.1136/bjo.65.4.226 7236565 PMC1039488

[B24] Helseth S Lund T Christophersen KA Health-related quality of life in a Norwegian sample of healthy adolescents: some psychometric properties of CHQ-CF87-N in relation to KINDL-N J Adolesc Health 2006 38 416 425 10.1016/j.jadohealth.2004.11.136 16549303

[B25] Ravens-Sieberer U Bullinger M Assessing health-related quality of life in chronically ill children with the German KINDL: first psychometric and content analytical results Qual Life Res 1998 7 399 407 9691720 10.1023/a:1008853819715

[B26] Yamaguchi N Poudel KC Poudel-Tandukar K Shakya D Ravens-Sieberer U Jimba M Reliability and validity of a Nepalese version of the Kiddo-KINDL in adolescents Biosci Trends 2010 44 178 185 20811137

[B27] Radloff LS The CES-D scale: a self report depression scale for research in the general population Appl Psychol Meas 1977 1 385 401 10.1177/014662167700100306

[B28] Rosenberg M Society and the adolescent self-image 1989 Middletown, Connecticut: Wesleyan University Press

[B29] Cohen J Statistical power analysis for the behavioral sciences 1988 2nd Hillsdale, NJ: Lawrence Earlbaum Associates

[B30] Rao AG Study of leprosy in children Indian J Lepr 2009 81 195 197 20704075

[B31] Nepal Central Bureau of Statistics Nepal living standard survey 2003/2004 2004 Kathmandu: Nepal Central Bureau of Statistics

[B32] Goodyear IM Developmental psychopathology: the impact of recent life events in anxious and depressed school-age children J R Soc Med 1994 87 327 329 8046702 10.1177/014107689408700609 PMC1294560

[B33] Nelson G Prilleltensky I MacGillivary H Building value-based partnerships: toward solidarity with oppressed groups Am J Community Psychol 2001 29 649 677 10.1023/A:1010406400101 11594694

[B34] Nepal South Asia Center Nepal human development report 1998 1999 Kathmandu: Nepal South Asia Center

[B35] World Health Organization World Health Report 2001: Mental health: New understanding, new hope 2001 Geneva: World Health Organization

[B36] Quinton D Rutter M Nicol AR Family pathology and child psychiatric disorder Longitudinal Studies in Child Psychology and Psychiatry: Practical Lessons from research experience 1985 Chichester: Wiley 91 134

[B37] Rotheram-Borus MJ Lee MB Gwadz M Draimin B An intervention for parents with AIDS and their adolescent children Am J Public Health 2001 91 1294 1302 10.2105/AJPH.91.8.1294 11499122 PMC1446764

